# *PICALM* Genetic Variant Alters mRNA Expression Without Affecting Protein Levels or Tau Spreading in Alzheimer’s Disease

**DOI:** 10.3390/cells15030235

**Published:** 2026-01-26

**Authors:** Kunie Ando, Lidia Lopez-Gutierrez, Salwa Mansour, Zehra Yilmaz, Luce Dauphinot, Jan Verheijen, Gaëlle Fontaine, Carolina Quintanilla-Sánchez, Emmanuel Aydin, Emilie Doeraene, Siranjeevi Nagaraj, Andreea-Claudia Kosa, Toshio Watanabe, Kristel Sleegers, Marie-Claude Potier, Jean-Pierre Brion, Karelle Leroy

**Affiliations:** 1 Alzheimer and Other Tauopathies Research Group, ULB Center for Diabetes Research, Medical Faculty, ULB Neuroscience Institute, Université Libre de Bruxelles (ULB), 808 Route de Lennik, B-1070 Brussels, Belgium; lidia.lopez.gutierrez@ulb.be (L.L.-G.); carolina.quintanilla.sanchez@ulb.be (C.Q.-S.); emmanuel.aydin@ulb.be (E.A.); emilie.doeraene@ulb.be (E.D.); siranjeevi.nagaraj@ulb.be (S.N.); andreea-claudia.kosa@ulb.be (A.-C.K.); jean-pierre.brion@ulb.be (J.-P.B.); 2 Laboratory of Histology, Neuropathology and Neuroanatomy, Faculty of Medicine, ULB Neuroscience Institute, Université Libre de Bruxelles, 808 Route de Lennik, B-1070 Brussels, Belgiumzehra.yilmaz@ulb.be (Z.Y.); 3 Sorbonne Université, Institut du Cerveau - Paris Brain Institute - ICM, CNRS, APHP, Hôpital de La Pitié-Salpêtrière, INSERM, F-75013 Paris, France; luce.dauphinot@cnrs.fr (L.D.); gaelle.fontaine@curie.fr (G.F.); marie-claude.potier@upmc.fr (M.-C.P.); 4 Complex Genetics of Alzheimer’s Disease Group, VIB Center for Molecular Neurology, VIB Antwerp, B-2610 Antwerp, Belgium; johannes.verheijen@aruplab.com (J.V.); kristel.sleegers@uantwerpen.vib.be (K.S.); 5 Department of Biomedical Sciences, University of Antwerp, B-2610 Antwerp, Belgium; 6 Department of Biological Science, Graduate School of Humanities and Sciences, Nara Women’s University, Nara 630-8506, Japan; toshiwatana@cc.nara-wu.ac.jp

**Keywords:** neurofibrillary tangles, paired helical filament, Alzheimer’s disease, argyrophilic grains, prion-like tau propagation, PICALM, tau, GWAS, qPCR, SNPs

## Abstract

Phosphatidylinositol-binding clathrin assembly protein (PICALM) is a clathrin adaptor essential for clathrin-mediated endocytosis. Genome-wide association studies (GWAS) have consistently identified *PICALM* as one of the most significant genetic susceptibility loci for late-onset sporadic Alzheimer’s disease (AD). However, the functional impact of the most validated AD-associated variant, rs3851179, remains unclear. Here, we examined *PICALM* mRNA and protein expression in *post-mortem* AD brains with reference to rs3851179 genotype. We found that *PICALM* mRNA levels were significantly increased in AD brains compared with controls, and that the protective rs3851179T allele was associated with reduced *PICALM* mRNA levels relative to the non-protective rs3851179C allele. In contrast, PICALM levels were significantly reduced in AD brain lysates compared with controls. PICALM expression did not significantly differ between carriers of the protective and non-protective alleles. Analysis of the mRNA-to-protein ratio revealed a significant dissociation between transcript and protein levels, suggesting relatively reduced protein expression efficiency in cases carrying the non-protective CC genotype. To assess whether reduced *PICALM* levels influence tau pathology, we used Picalm heterozygous knockout (Picalm+/−) mice, which express approximately 50% of the wild-type Picalm protein. Following stereotaxic injection of pathological tau extracted from AD brains, both wild-type and Picalm+/− mice developed tau pathology; however, the extent of tau accumulation did not significantly differ between genotypes. Together, these findings indicate that although PICALM protein level is reduced in AD, this reduction does not appear to affect tau propagation in this model. Therefore, the AD susceptibility associated with *PICALM* variant likely arises from mechanisms other than tau spread, possibly involving other aspects of autophagy, endocytic or vascular function.

## 1. Introduction

Alzheimer’s disease (AD) is the most common form of dementia and has become a major public health concern in aging societies [1,2,3]. AD is neuropathologically defined by two hallmark lesions: extracellular amyloid plaques and intracellular neurofibrillary tangles (NFTs). NFTs are composed of paired helical filaments (PHFs) formed from hyperphosphorylated and misfolded tau protein [4,5,6]. Amyloid plaques consist of aggregated amyloid-β (Aβ) peptides surrounded by tau-positive dystrophic neurites and reactive glial cells [7]. Among these two pathologies, NFTs show a stronger correlation with cognitive decline and are now considered reliable biomarkers and therapeutic targets for AD [8,9,10]. Aβ, inflammatory, vascular, and infectious mechanisms have interactions in AD pathophysiology [11].

Tau pathology occurs not only in AD but also in numerous tau-related neurodegenerative diseases, so-called tauopathies, such as Pick’s disease, familial frontotemporal dementia and parkinsonism linked to chromosome 17 (FTDP-17) [12], progressive supranuclear palsy (PSP) [13], corticobasal degeneration (CBD) [14], and argyrophilic grain disease (AGD) [15], among others [4]. In AD, tau aggregates spread along anatomically connected brain regions [16]. Increasing evidence supports a prion-like mechanism of tau propagation, in which misfolded tau spreads from neuron to neuron via synaptic connections [17,18,19]. Neuropathological studies have shown that NFT pathology first appears in the locus coeruleus [20], then progresses through the transentorhinal cortex, limbic system, and ultimately reaches widespread cortical areas [21,22]. This propagation can be experimentally induced by intracerebral injection of AD brain extracts [23,24,25] or purified AD-derived PHFs (AD-PHF) [26,27,28]}.

Genetic studies have identified several AD susceptibility loci in the *APOE*, *PICALM*, *BIN1*, and *CD2AP* genes [29,30,31,32,33,34,35,36,37,38,39,40,41,42,43,44]. These genes encode proteins involved in clathrin-mediated endocytosis (CME), a pathway implicated in AD pathogenesis [45,46,47]. Both *APOE* and *PICALM* are major genetic risk factors for AD that converge on lipid metabolism and endocytic trafficking pathways, thereby influencing Aβ clearance and tau pathology [48]. PICALM has been shown to colocalize with endothelial cell markers [49,50,51] and hyperphosphorylated tau in NFTs [52], and its expression is reduced in the soluble fractions of AD and other tauopathy brain tissues [53]. Moreover, PICALM reduction exacerbates tau pathology in a transgenic mouse model expressing human tau carrying FTDP-17 mutations [54]. Despite these findings, the role of PICALM in the prion-like propagation of tau pathology remains unclear.

In this study, we investigated whether the AD-protective variant rs3851179T influences *PICALM* expression and tau propagation. Next, we assessed the effect of Picalm reduction on tau propagation in vivo using a stereotaxic injection model, in which AD-derived PHFs (AD-PHF) were injected into the hippocampus of wild-type and Picalm heterozygous (Picalm+/−) mice [55].

## 2. Materials and Methods

### 2.1. Human Brain Tissues

Frozen samples from the superior temporal T1 isocortex were obtained from individuals with AD and age-matched non-demented control subjects [56]. Control cases were defined as individuals without dementia who died without any known neurological disorders. AD cases were diagnosed according to the National Institute of Aging and Reagan Institute Criteria [57,58] and were scored by neuropathological staging of tau and amyloid pathologies (Braak and Thal staging, respectively) [16,59]. AD cases, including two familial AD (FAD) cases carrying *Amyloid Precursor Protein* (*APP*) or *Presenilin1* (*PSEN1*) mutations, were all scored as Braak’s stage V or VI (Supplementary Table S1). The mean ages and post-mortem delays of control and AD cases were not significantly different. Average age at death was 77.37 +/− 10.00 years for controls (*n* = 41) and 75.80 +/− 10.43 years for AD cases (*n* = 51) (mean +/− SD; *p* = 0.4). Average *post-mortem* delays were 23.33 +/− 14.20 h and 20.27 +/− 12.46 h for control and AD cases (mean +/− SD; *p* = 0.4) respectively. *Apolipoprotein E* (*ApoE*) genotype was determined for cases with informed consent for genetic study by PCR amplification of genomic DNA followed by sequencing, as previously described [60]. *PICALM* rs3851179 genotype of the same individuals was determined on genomic DNA by PCR amplification followed by Sanger sequencing using the BigDye termination cycle sequencing kit v3.1 on the ABI 3730 DNA Analyzer (Thermo Fisher Scientific, Gosselies, Belgium). Sequences were analyzed using Seqman (DNAstar, Madison, WI, USA) and NovoSNP software (version 2017) [61].

### 2.2. RNA Extraction and Quantitative PCR (qPCR) for PICALM mRNA

Total RNAs from human T1 or frontal isocortex were extracted using Nucleospin RNA II kit (Macherey-Nagel, Duren, Germany). The quality and quantity of each RNA were assessed with an Agilent 2100 Bioanalyzer using RNA 6000 NanoChips (Agilent Technologies, Santa Clara, CA, USA). Briefly, 500 ng of RNA was reverse-transcribed into cDNAs (10 min at 25 °C, then 2 h at 42 °C and 5 min at 85 °C) using the SensiFAST cDNA synthesis kit (Bioline-Meridian Bioscience, London, UK) according to the manufacturer’s instructions. qPCR assays were performed on a LightCycler 96 system (Roche, Boulogne-Billancourt, France) using 1× LightCycler^®^ 480 Probes Master mix (Roche, Boulogne-Billancourt, France), 200 nM of each primer, and 100 nM of specific hydrolysis probe (designed with the Universal Probe Library, Roche Applied Science). Our analyses focused on the major brain isoforms of *PICALM*, including NM_007166.3, NM_001008660.2, and NM_001206946.1. The detected isoforms analyzed represent the predominant PICALM transcripts detected in adult human brain and account for the majority of *PICALM* mRNA abundance [50,62,63]. *PICALM* mRNA expression was normalized to the average of the three stable controls, *Peptidylprolyl Isomerase B* (*pPib*), *Ring Finger Protein 4* (*RNF4*), and *DNA-directed RNA polymerase II subunit RPB1* (*PolR2A*) [64]. The following primers were used:

*PICALM*: forward 5′ ctgaccaaagtggatgaaagg 3′; reverse 5′ ttcttttaggcgctgttcct 3′;

*PolR2A*: forward 5′ caagttcaaccaagccattg 3′; reverse 5′ gtggcaggttctccaagg 3′;

*pPib*: forward 5′ ttcttcataaccacagtcaagacc 3′; reverse 5′ accttccgtaccacatccat 3′;

*RNF4*: forward 5′ctcaggtactgtcagttgtc 3′; reverse 5′cgatgagacgtccattctg 3′.

### 2.3. Preparation of Brain Homogenates for Biochemical Analysis

About 200 mg of frozen T1 or frontal isocortex was homogenized as previously reported [52,65] in 10 volumes of ice-cold RIPA buffer containing 50 mM Tris-HCl (pH 7.4), 50 mM NaCl, 1% NP-40, 0.25% sodium deoxycholate, 5 mM EDTA, 1 mM EGTA, protease inhibitor cocktail (Sigma-Merck, Brussels, Belgium, 11697498001), 1 mM PMSF (Sigma-Merck, P-7626), and phosphatase inhibitor cocktail 2 (Sigma-Merck, P-5726). The samples were incubated for 60 min at 4 °C on a rotator. The homogenate was centrifuged (20,000× *g* for 20 min, 4 °C) and the supernatant was collected as the RIPA-soluble fraction. Protein concentrations were determined by the Bradford method (Bio-Rad, Nazareth, Belgium, 5000205), and 25 µg of protein per sample was loaded for SDS-PAGE.

### 2.4. Antibodies

The rabbit polyclonal anti-PICALM HPA019053 antibody was purchased from Sigma-Merck. This antibody reacts with both human and mouse PICALM [66]. Mouse monoclonal anti-actin (A-5441) was also purchased from Sigma-Merck. Mouse monoclonal PHF1 antibody was provided by Dr. Peter Davies and recognizes pSer396/404 of tau [67]. The rabbit polyclonal anti-total tau B19 antibody was raised to adult bovine tau proteins and reacts with all known adult and fetal tau isoforms in bovine, rat, mouse, and human nervous tissue in a phosphorylation-independent manner [68].

### 2.5. Western Blot (WB)

Tissue lysates were run in 7.5% Tris-Glycine gels and transferred onto nitrocellulose membranes (sc-3724, Santa Cruz Biotechnology, Heidelberg, Germany). Membranes were blocked in 10% (*w*/*v*) semi-skimmed milk in TBS (Tris-HCl 0.01 M, NaCl 0.15 M, pH 7.4) for 1 h at room temperature, incubated with primary antibodies overnight, and then with horseradish peroxidase (HRP)-conjugated secondary antibodies (anti-rabbit #7074, Cell Signaling Technology, Bioké, Leiden, The Netherlands; anti-mouse A-6782, Sigma-Merck). After washing, membranes were developed with SuperSignal West Pico PLUS Chemiluminescent Substrate (Thermo Fisher Scientific) and imaged using a DARQ-7 CCD camera (Vilber-Lourmat, Marne-la-Vallée, France) in a SOLO 4S WL system. The optical density (OD) of protein signals was quantified by densitometry analysis using the NIH ImageJ program (version 1.53a), and actin was used as a loading control.

### 2.6. Preparation of Human Sarkosyl-Insoluble PHF-Tau Fraction

Sarkosyl extraction was performed as previously described [68,69,70]. Briefly, 0.5 g of frozen frontal cortex gray matter from control (Braak I, Thal 0) and AD (Braak V-VI, Thal 4) cases was homogenized in 10 volumes of ice-cold PHF-extraction buffer (10 mM Tris-HCl, pH 7.4; 0.8 M NaCl; 1 mM EDTA; 10% sucrose). Homogenates were centrifuged at 20,000× *g* for 20 min at 4 °C. The supernatant was collected, and the pellet was re-homogenized and centrifuged under the same conditions. Supernatants were pooled, and N-lauroylsarcosine sodium salt (L-5125; Sigma-Merck) was added to reach a final concentration of 1% (*w*/*v*). The samples were incubated overnight at 4 °C with mild agitation, followed by an ultracentrifugation at 180,000× *g* for 30 min at 4 °C. The Sarkosyl-soluble supernatant was discarded, and the Sarkosyl-insoluble pellet was briefly rinsed and re-suspended in 0.25 mL of phosphate-buffered saline (PBS, pH 7.4) by vigorous pipetting. The Sarkosyl-insoluble PHF-tau fraction was sonicated on ice at amplitude 60 for 40 impulsions of 1 s, with 10 s interval after each 10 pulses. Protein concentration was determined using the Bradford assay (Bio-Rad) and adjusted to 1 mg/mL. Sarkosyl-insoluble fractions were aliquoted and stored at −20 °C, and were subsequently analyzed by WB and transmission electron microscopy (TEM) as previously described [64,65].

### 2.7. Mouse Line

The Picalm+/− line was generated by inserting PGK-neo-pA into the first exon of *Picalm* gene [55]. Hemizygous Picalm+/− mice were maintained on a C57BL/6J background by backcrossing for at least five generations. Genotyping was performed by 2 independent polymerase chain reaction (PCR) amplifications of DNA extracted from ear biopsies using the following primers:

-Primer A: forward 5′-ATG TCT GGC CAG AGC CTG ACG GAC CGA ATC-3′ and C: reverse 5′-GGG TCG GGA GAG GAT GCG GGG GGT CTT CAC-3′) for wild-type allele. -Neogt-1: reverse 5′-CTG ACC GCT TCC TCG TGC TTT ACG-3′ for the knockout allele [55]. 

All animal studies were performed in compliance with the ethical guidelines and approved by the Ethical Committee for the Care and Use of Laboratory Animals at the Medical School of the Free University of Brussels.

### 2.8. Stereotaxic Injection

Nine-month-old female wild-type and Picalm+/− mice were used. A total of 50 mice were injected: 10 wild-type and 10 Picalm+/− mice received Sarkosyl-insoluble fractions from control brain, while 13 wild-type and 17 Picalm+/− mice were injected with AD-PHF. Mice were deeply anesthetized by intraperitoneal injection of a ketamine/xylazine mixture prepared in physiological saline (ketamine hydrochloride 100 mg/kg; Nimatek, Vetoquinol; xylazine 10 mg/kg; Rompun, Bayer; injection volume: 100 µL per 10 g body weight), as previously described [26,71]. Mice were then placed in a stereotaxic frame (Kopf Instruments, Düsseldorf, Germany). Unilateral stereotaxic injections were performed into the left hippocampus at the level of the dentate gyrus using the following coordinates relative to bregma: anteroposterior −2.1 mm, mediolateral +1.5 mm, dorsoventral −2.0 mm. Sarkosyl-insoluble material (1 µg) from either control or AD brain was injected in a total volume of 1 µL (final concentration: 1 µg/µL) at a rate of 0.2 µL/min using a microinjection pump (KD Scientific, Holliston, MA, USA) and a 200 µm-diameter needle (Thermo Fisher Scientific). The needle was left in place for 5 min after completion of the injection to allow diffusion of the material and to minimize reflux, and was then slowly withdrawn.

### 2.9. Spatial Memory Test

Wild-type and Picalm+/− mice injected with control or AD-PHF fractions were evaluated in the Y-maze test three months after injection. The task began with a 5 min habituation phase during which one arm of the maze (the “novel arm”) was closed. After a 2 min intertrial interval, mice were allowed to freely explore all three arms of the maze for 4 min. Spatial memory was assessed by quantifying the time spent in the novel arm during the first minute of the exploration phase [72].

### 2.10. Immunohistochemistry

After formaldehyde fixation (10% buffered formalin), brain tissues were paraffin-embedded and sliced into 7 µm thick sections. Staining by 3,3′-diaminobenzidine (DAB) was performed as previously described [52], and the sections were examined under a Leica DM500 microscope (Leica, Nanterre, France). In the histological sections, the presence of tau-positive granular structures around the injection site was carefully verified and analyzed in the PHF-injected mice.

For quantitative analysis, tau-positive structures in the hippocampus of wild-type and Picalm+/− mice were analyzed from 40× images using thresholding analyses in NIH ImageJ, as previously reported [71,73].

### 2.11. Statistical Analyses

Sample sizes are indicated in the figure legends. Statistical analyses and normality tests were performed using GraphPad Prism 9. Depending on the data distribution and experimental design, group comparisons were carried out using unpaired two-tailed Student’s *t*-tests, Mann–Whitney U tests, Spearman’s correlation test, one-way ANOVA, two-way ANOVA or Kruskal–Wallis tests, as specified in the figure legends. Data are presented as mean +/− SEM (standard error of the mean). A *p*-value < 0.05 was considered statistically significant.

## 3. Results

### 3.1. PICALM Expression in Human Brains

#### 3.1.1. Expression of *PICALM* mRNA Is Increased in Human AD Brains Compared to Non-Demented Control Brains

**Figure 1:**
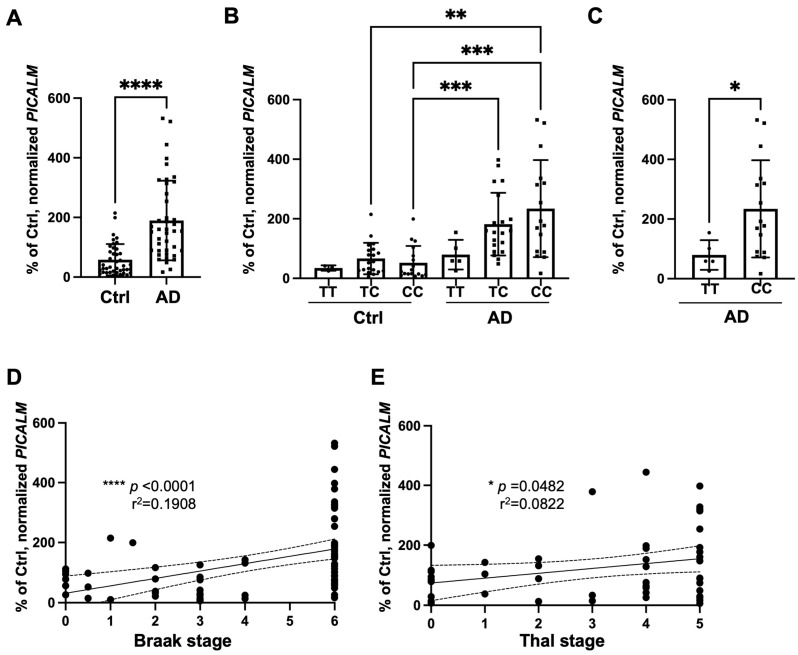
The analyses of *PICALM* mRNA expression in the *post-mortem* human brains. (**A**) The normalized *PICALM* mRNA was significantly increased in AD brains compared to non-demented control brains. **** *p* < 0.0001 by Mann–Whitney U test for control (*n* = 40) and AD (*n* = 41) cases. (**B**) The non-protective rs3851179C allele was associated with an upregulated *PICALM* mRNA expression in the AD cohort. There was a significant difference detected by Kruskal–Wallis test followed by Dunn’s post hoc test (** *p* < 0.01, *** *p* < 0.001) for control bearing TT (n = 3), control bearing TC (*n* = 20), control bearing CC (*n* = 16), AD bearing TT (*n* = 5), AD bearing TC (*n* = 20), and AD bearing CC (*n* = 16) at rs3851179. (**C**) There was a significant difference in *PICALM* mRNA levels among AD cases with the protective TT allele and those with the non-protective CC. * *p* < 0.05 by Mann–Whitney U test. (**D**,**E**) There is a significant correlation between normalized *PICALM* mRNA and Braak stages (*n* = 83) or Thal stages (*n* = 49) by Spearman correlation tests. 95% confidence bands are shown in a dashed line.

We first analyzed the *PICALM* mRNA levels in non-demented control and AD brains regardless of rs3851179. We found a significant increase in *PICALM* mRNA levels in AD brain samples compared with controls (Figure 1A).

We then examined *PICALM* expression in relation to protective rs3851179T versus non-protective rs3851179C alleles. *PICALM* mRNA expression was markedly increased in AD cases carrying the non-protective CC genotypes (Figure 1B). When the two groups of protective (TT) and non-protective (CC) cases were compared among AD cases, a significant difference was observed, suggesting lower *PICALM* mRNA expression in AD cases with the protective TT genotype compared with those carrying the non-protective CC genotype (Figure 1C).

There was a modest but significant correlation between *PICALM* mRNA and Braak stages or Thal stages (Figure 1D,E). We also analyzed other confounding factors, including age, PMI, sex, and *APOE* genotype (Supplementary Figure S1). No significant associations were detected with these factors, whereas the rs3851179 allelic variant showed a robust effect, with the non-protective C allele significantly associated with increased *PICALM* mRNA levels.

#### 3.1.2. PICALM Is Decreased in AD Brains, and Its Level Is Not Significantly Affected by rs3851179 Genotype

**Figure 2:**
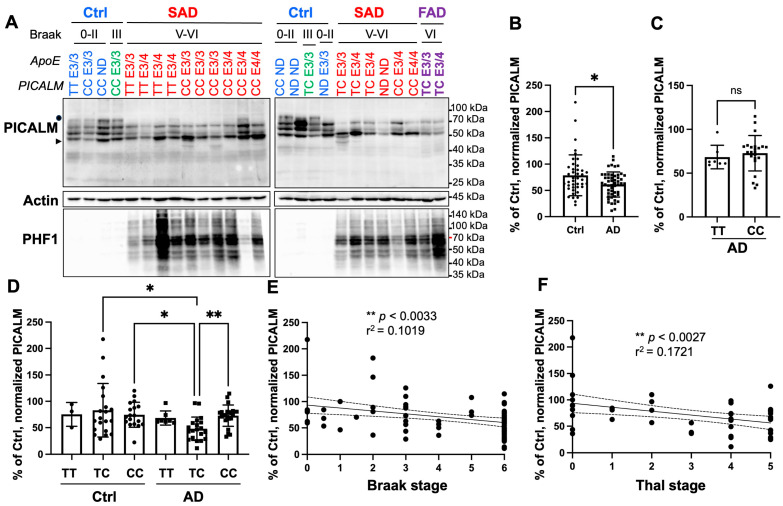
WB analyses of PICALM expression in the *post-mortem* human brains. (**A**) Representative images showing WB analyses of PICALM, actin, and pTau using PHF1 are shown. The RIPA-soluble fraction was analyzed. (**B**) The expression of PICALM normalized to actin was significantly decreased in AD brains compared to non-demented controls. The reduction in the longest PICALM isoform (shown in a black circle) was most prominent. Some AD samples contained proteolyzed PICALM fragments detected below 50 kDa (shown in black arrowhead). * *p* < 0.05 by Mann–Whitney U test for control (*n* = 41) and AD (*n* = 51) cases. (**C**) There was no significant difference in normalized PICALM level when the AD samples were divided into TT versus CC alleles and were analyzed by the Mann–Whitney U test (*n* = 7 for control and *n* = 23 for AD cases). (**D**) PICALM levels were significantly reduced in AD-TC cases compared to Ctrl-TC, Ctrl-CC, or AD-CC cases by the Kruskal–Wallis test (*n* = 3 for Ctrl-TT, *n* = 20 for Ctrl-TC, *n* = 18 for Ctrl-CC, *n* = 7 for AD-TT, *n* = 20 for AD-TC and, *n* = 23 for AD-TT at rs3851179). * *p* < 0.05, ** *p* < 0.01. (**E**,**F**) There were significant inverse correlations between PICALM levels and Braak stages ((**E**), *n* = 81) or Thal stages ((**F**), *n* = 48) by Spearman correlation test (** *p* < 0.01). Uncropped WB images with molecular weight markers are shown in Supplementary Figure S2. 95% confidence bands are shown in a dashed line. ND, not determined.

We next analyzed PICALM levels by WB (Figure 2A). Consistent with previous findings [51,52], PICALM levels were significantly decreased in AD brains compared with controls (Figure 2A,B; Supplementary Figure S2). Notably, the longest isoform band was generally decreased in AD brains, and protein degradation was confirmed by the presence of shorter fragments in some AD samples (Figure 2A).

However, PICALM levels did not differ significantly between AD cases carrying the protective TT and non-protective CC genotypes at rs3851179 (Figure 2C). When all the genotypes are compared, the reduction in PICALM in AD cases carrying the TC genotype was most remarkable. There was a significant reduction in AD cases with TC genotypes compared to control cases with TC or CC genotypes and AD cases with CC genotypes (Figure 2D). The protein levels of PICALM negatively correlated with both Braak and Thal stages (Figure 2E,F).

Moreover, no significant interactions were observed between PICALM levels with age, PMI, sex, and *APOE* genotype (Supplementary Figure S3).

**Figure 3:**
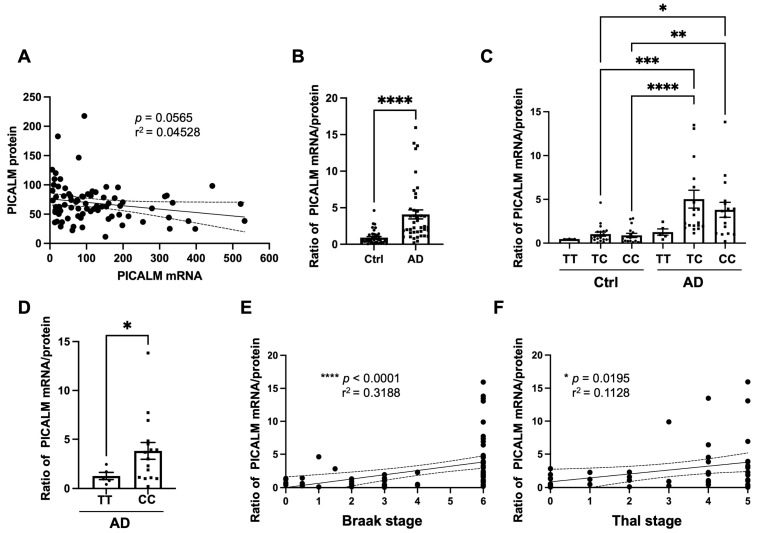
The ratio of *PICALM* mRNA to PICALM protein levels, as estimated by WB, indicates an excess of mRNA relative to the amount of detectable PICALM protein. (**A**) There was no significant correlation between *PICALM* mRNA and protein levels. (*n* = 81, *p* = 0.0565, r^2^ = 0.04528 by Spearman correlation test). (**B**) There was a significant difference in the ratio of *PICALM* mRNA to PICALM between control and AD brains. In AD brains, there is proportionally abundant *PICALM* mRNA compared to controls. *n* = 40 for control and *n* = 41 for AD cases and **** *p* < 0.0001 by Mann–Whitney U test. (**C**) There was a general increase in the ratio of *PICALM* mRNA/protein in the AD cases carrying one or two C alleles at rs3851179 compared to control cases or AD cases carrying the protective TT allele at rs3851179. * *p* < 0.05, ** *p* < 0.01, *** *p* < 0.001, **** *p* < 0.0001 by Kruskal–Wallis test. (**D**) There was a significant difference between AD cases bearing TT and those CC at rs3851179. * *p* < 0.05 by Mann–Whitney U test (*n* = 5 for AD-TT and *n* = 16 for AD-CC). (**E**,**F**) There were significant correlations between *PICALM* mRNA/protein ratio and Braak stages ((**E**), *n* = 83) or Thal stages ((**F**), *n* = 50). * *p* < 0.05 and **** *p* < 0.0001 by Spearman correlation tests. 95% confidence bands are shown in a dashed line.

It was surprising to observe such a clear discrepancy between *PICALM* mRNA and protein data, showing that *PICALM* mRNA was upregulated in AD brains while the protein level was significantly decreased. Moreover, higher *PICALM* mRNA observed in CC allele carriers was not reproduced as altered PICALM expression by WB between these two groups, suggesting no significant correlation between *PICALM* mRNA and protein (Figure 3A).

The dissociation between *PICALM* mRNA and protein expression became even more evident when the ratio of *PICALM* mRNA to PICALM was analyzed (Figure 3B–D). The *PICALM* mRNA/protein ratio was significantly higher in AD brains compared with control brains (Figure 3B). Furthermore, this ratio was generally higher in AD cases carrying one or two non-protective alleles (TC or CC) than those with the protective TT genotype (Figure 3C). Comparison of TT versus CC in AD cases using the Mann–Whitney test confirmed a significant difference between the two groups (Figure 3D).

Interestingly, the *PICALM* mRNA/protein ratio correlated with Braak and Thal stages (Figure 3E,F). Taken together, these data suggest that while *PICALM* mRNA levels are more elevated in AD brains relative to controls, PICALM levels decrease in association with Braak and Thal stages. This dissociation between transcription and translation appears to be related both to disease progression and to genetic variation at rs3851179.

These findings suggest that *PICALM* mRNA translation may be less efficient in AD brains than in controls. Moreover, the non-protective C allele may further impair this translational process, as reflected by the significant increase in the mRNA/protein ratio in AD cases carrying the CC genotype compared with those carrying the TT genotype.

### 3.2. Stereotaxic Injection of AD-PHF into Wild-Type and Picalm+/− Mice Suggests No Significant Change in Tau Pathology Propagation When Picalm Expression Is Reduced by 50%

#### 3.2.1. Characterization of AD-PHF and Stereotaxic Injection to Wild-Type and Picalm+/− Mice

Our WB data from human control and AD brains showed a significant reduction in Picalm levels, suggesting a potential impairment in the translation of *PICALM* mRNA into protein. We therefore hypothesized that reduced PICALM expression may contribute directly to AD pathogenesis. Since PICALM is a key component of clathrin-mediated endocytosis, a process implicated in pathological tau seeding and propagation [45,46], we postulated that PICALM might regulate AD susceptibility through mechanisms related to tau propagation.

**Figure 4:**
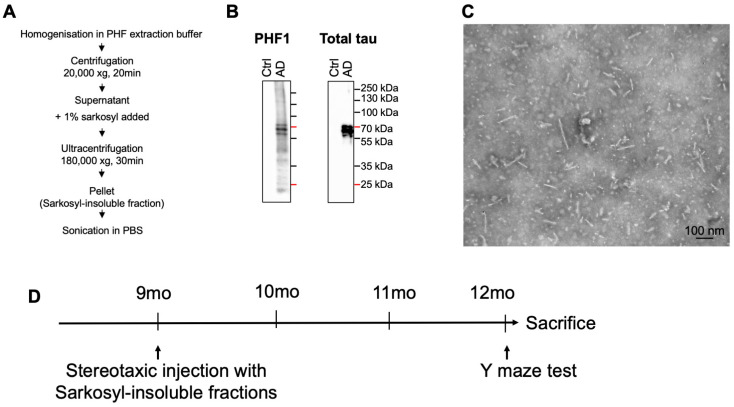
Characterization of Sarkosyl-insoluble fractions prepared from control and AD *post-mortem* human brains. (**A**) A simplified schematic illustration of the preparation of the Sarkosyl-insoluble fraction. (**B**) WB analysis showed the presence of PHF1-positive pTau (pSer396/Ser404) and total tau in the Sarkosyl-insoluble fraction prepared from human *post-mortem* AD brains. In the same fraction obtained from control brains, there was no PHF-tau detected. (**C**) A representative image of the Sarkosyl-insoluble fraction analyzed in transmission electron microscopy (TEM). Scale bar, 100 nm. (**D**) A timeline of the stereotaxic injection (9 months) and incubation (from 9 to 12 months), then behavioral test and sacrifice. Uncropped WB images merged with molecular weight markers are shown in Supplementary Figure S4.

To test this hypothesis, we used a stereotaxic injection model of PHF-tau propagation [24,26,27,74]. Following our established protocol [68], Sarkosyl-insoluble fractions were extracted from control (non-demented) and AD brains (Figure 4A). As previously described using denaturing SDS-PAGE [26], PHF1-positive hyperphosphorylated tau was present in the Sarkosyl-insoluble fraction from AD brains as three major species of 57–69 kDa, but absent in control samples (Figure 4B; Supplementary Figure S4). TEM analyses confirmed the presence of PHF in AD-derived fractions (Figure 4C), which are known to induce tau-positive argyrophilic inclusions when injected into wild-type mouse brains [26,71].

The Sarkosyl-insoluble fractions from AD and control brains were injected intracerebrally into the dentate gyrus of the left hippocampus of wild-type and Picalm+/− mice. Because Picalm−/− mice are lethal within one month after birth [55], they were not included in this study. Picalm+/− mice, however, displayed no macroscopic or behavioral abnormalities up to 12 months of age [54].

9-month-old wild-type and Picalm+/− mice were unilaterally injected with 1 µg of either control or AD-PHF fractions into the hilus of the dentate gyrus. The mice were analyzed by Y maze test for working memory and sacrificed at 12 months, 3 months post-injection (PI), for histological and biological analyses (Figure 4D).

#### 3.2.2. Working Memory Was Not Significantly Affected by AD-PHF Injection in Wild-Type and Picalm+/− Mice

**Figure 5:**
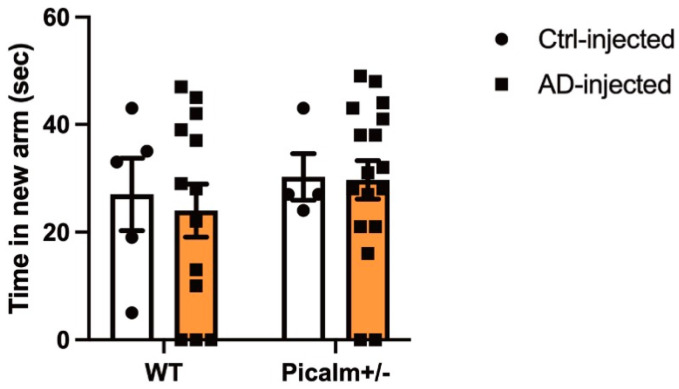
Spatial memory is unchanged between AD-PHF–injected wild-type and Picalm+/− mice. Three months after intracerebral injection of AD-PHF, no significant difference in the time spent in the novel arm of the Y-maze was observed between wild-type and Picalm+/− mice. Wild-type (*n* = 5) and Picalm+/− mice (*n* = 4) injected with control Sarkosyl-insoluble fractions were used as controls. Wild-type (*n* = 13) and Picalm+/− mice (*n* = 17) received AD-PHF injections. No significant group differences were detected by two-way ANOVA. WT, wild-type. Data are presented as mean +/− SEM.

To assess the functional consequences of AD-PHF injection in wild-type and Picalm+/− mice, we performed a Y maze test equipped with a door to evaluate spatial learning and memory at 3 months PI. No significant difference was observed between AD-PHF-injected wild-type and Picalm+/− mice in the time spent exploring the novel arm (Figure 5). These results suggest that a 50% reduction in Picalm expression did not affect memory functions compared with wild-type counterparts injected with AD or control fractions.

#### 3.2.3. No Significant Change in Prion-like Tau Pathology Propagation in Picalm+/− Mice

**Figure 6:**
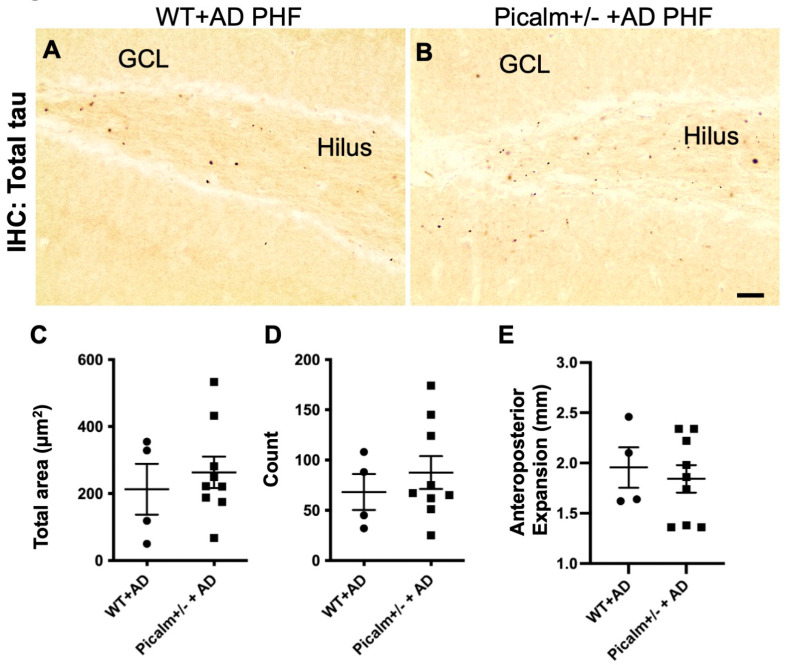
No significant difference was observed in tau pathology progression between wild-type and Picalm+/− mice injected with AD-PHF. (**A**,**B**) Representative images of tau-positive granular structures observed near the injection sites of the hilus of sex-matched (female) wild-type (**A**) and Picalm+/− (**B**) mice 3 months after the intracerebral injection of AD-PHF. Scale bar, 40 µm. (**C**,**D**) Quantification of the total area (**C**) and total number (**D**) of tau-positive structures observed in the hilus. The section adjacent the injection site was analyzed. (**E**) The anteroposterior propagation of tau pathology was analyzed in histological sections. There was no significant difference between AD-PHF-injected wild-type (*n* = 4) and Picalm+/− (*n* = 9) (*p* > 0.05 by unpaired Student *t*-test). WT, wild-type.

AD-PHF prepared from AD brains induced grain-like tau positive inclusions in wild-type mouse brains (Figure 6A), whereas control samples did not cause any detectable tau pathology at 3 months PI (Supplementary Figure S5A,B) [26]. Numerous tau-positive granular structures were observed in the hilus and granule cells of the hippocampus in both AD-PHF-injected wild-type and Picalm+/− mouse brains at 3 months PI (Figure 6A,B).

To investigate whether Picalm reduction influences the induction of tau pathology, we examined the spatiotemporal spread of tau-positive grains immunolabelled by anti-total tau B19 antibody in AD-PHF-injected wild-type and Picalm+/− mice. No significant differences were observed in the total area occupied by tau labeling or the number of tau-positive structures at the injection sites between AD-PHF-injected wild-type and Picalm+/− mice (Figure 6C,D). Consistently, analysis of anteroposterior tau propagation indicated no significant difference between AD-PHF-injected wild-type and Picalm+/− mice (Figure 6E).

These data suggest that reducing Picalm expression by 50% does not significantly affect prion-like tau propagation in this model.

**Figure 7:**
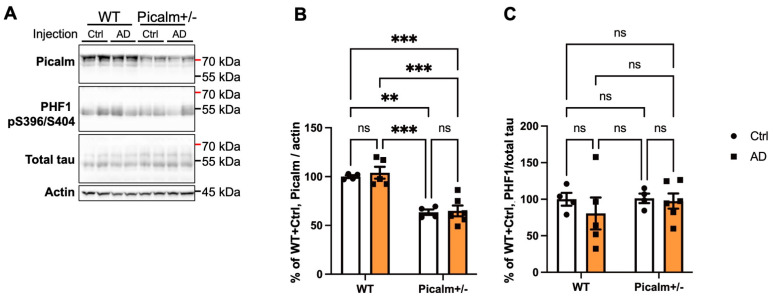
There was no clear difference in pTau levels detected by PHF1 antibody in the brain lysates of wild-type (WT) or Picalm+/− mice injected with AD-PHF. (**A**) Representative images of WB analyses for Picalm, pTau (PHF1), total tau, and actin of the sex-matched (female) brain lysates. Mice were injected with the Sarkosyl-insoluble fractions from either control or AD cases and were analyzed at 3 months PI. (**B**) Quantification of actin-normalized Picalm shows that there was an approximately 50% of reduction in the Picalm expression level in Picalm+/− mouse brains. (**C**) Quantification of PHF1-positive tau normalized to total tau suggests that there was no clear difference between AD-injected wild-type and Picalm+/− mice. Two-way ANOVA with Šidák’s multiple comparisons test was performed for (**B**,**C**). **: *p* < 0.01, ***: *p* < 0.001, ns: not significant. Wild-type (*n* = 4) and Picalm+/− mice (*n* = 4) injected with control Sarkosyl-insoluble fractions were used as controls. Wild-type (*n* = 5) and Picalm+/− mice (*n* = 7) received AD-PHF injections.

We also compared Picalm and pTau levels in brain lysates from wild-type and Picalm+/− mice injected with AD-PHF (Figure 7A, Supplementary Figure S6). First, we confirmed that Picalm expression was reduced by approximately 50% in Picalm+/− mouse brains compared with wild-type littermates (Figure 7B), consistent with previous reports [54,55]. We then analyzed the pTau level using the PHF1 antibody, which recognizes pSer396/pS404 tau [67]. No significant differences in pTau levels were detected among control- or AD-PHF-injected wild-type and Picalm+/− mice (Figure 7C).

Taken together, these results from our stereotaxic injection model, with a post-injection interval in 9–12-month-old mice, suggest that Picalm reduction does not influence tau pathology propagation in mouse brains.

## 4. Discussion

In this study, we demonstrated that *PICALM* mRNA levels were elevated in AD brains compared with those in cognitively healthy controls. Moreover, AD cases carrying the protective T allele exhibited lower *PICALM* mRNA expression than those carrying the non-protective C allele at rs3851179. In contrast, PICALM levels were significantly reduced in AD brain lysates compared with controls, and no difference was observed between TT- and CC- genotype carriers. Calculation of the *PICALM* mRNA-to-protein ratio revealed a significant dissociation between transcript and protein levels in AD brains, particularly between AD cases bearing the protective and non-protective alleles.

Interestingly, our findings contrast with those of Zhao et al. (2015), who reported that iPSC-derived human endothelial cells carrying the protective TT allele at rs3851179 showed increased *PICALM* expression and enhanced Aβ clearance [51]. The discrepancy between their observation and ours may arise from cell-type-specific differences. While Zhao et al. analyzed endothelial cells, our data were obtained from bulk isocortex gray matter, which contains multiple cell types (neurons, microglia, astrocytes, endothelial cells, etc.). In such bulk tissue, potential allele-specific expression differences may be masked, consistent with previous reports showing no clear allelic expression imbalance with rs3851179 [75].

Indeed, recent expression quantitative trait locus (eQTL) studies have shown that the effect of *PICALM* variants is highly cell-type dependent, particularly in microglia [63,76,77,78,79]. *PICALM* rs10792832, which is in almost complete linkage disequilibrium with rs3851179, lies in an open chromatin region, and the non-protective variant has been associated with both reduced chromatin accessibility and lower gene expression [63,77]. Microglia carrying the non-protective allele exhibited reduced *PICALM* expression, lipid droplet accumulation, and phagocytosis deficits [63]. Thus, our observations in mixed brain tissue may reflect the averaged contributions of multiple cell types, potentially masking cell-specific effects of rs3851179.

Our results are consistent with the study by Baig et al., who also reported increased *PICALM* mRNA levels in AD brains [49], and with our previous finding of reduced PICALM levels in AD [52]. Discrepancies between mRNA and protein abundance are commonly reported in human brain studies [80], likely reflecting post-transcriptional or translational regulation, altered protein stability, or degradation [81]. Indeed, PICALM is a protein particularly vulnerable to proteases such as calpain or caspases [82,83] abnormally activated in AD brains [84,85].

Together, these findings support the hypothesis that rs3851179 regulates *PICALM* transcription [86], and that the AD state and the non-protective C allele may further exacerbate the dissociation between transcript and protein expression. The observed dissociation between mRNA and protein levels may also reflect post-transcriptional regulatory mechanisms, including differences in mRNA stability or alternative splicing, which were not directly assessed in this study. In addition, post-translational processes such as phosphorylation, altered intracellular trafficking, or enhanced protein degradation could further influence PICALM abundance, thereby contributing to the mismatch between transcript levels and the final protein product. Finally, as the primers used here target the major brain isoforms of *PICALM*, we cannot exclude disease-associated changes in low-abundance or cell-type-restricted *PICALM* transcripts that may not be captured by our analysis.

Recent studies have highlighted the potential role of circular RNAs (circRNAs), including those derived from *PICALM*, in the regulation of gene expression and cellular processes [87,88]. CircRNAs are often regulated independently of their linear counterparts and have been implicated in the modulation of protein synthesis and disease mechanisms. Notably, circRNAs may contribute to discrepancies observed between mRNA and protein abundance, which could provide additional insights into the roles of *PICALM* in AD [89]. Although our study focused on linear *PICALM* expression and its effects on tau pathology, the contribution of circRNAs to these processes represents an exciting avenue for future research.

Moreover, alternative splicing of *PICALM* and its binding partners (*AP2A2* and *AP2A1*) has been shown to change during AD progression [90]. Interestingly, *Microtubule-associated protein tau* (*MAPT*) expression can modulate *PICALM* splicing in iPSC-derived neurons, suggesting that tau accumulation could influence RNA processing of genes involved in receptor-mediated endocytosis and autophagy [86]. Such interactions may partly explain the altered *PICALM* mRNA-protein balance we observed in AD brains.

While the murine model provides valuable insights into the functional consequences of reduced Picalm expression, the model does not fully recapitulate the human regulatory landscape, including genetic and age-related factors. The mouse data serve as a useful starting point for understanding the potential effects of reduced Picalm expression, but they should not be directly equated with the complex regulatory mechanisms of human disease contexts.

In our stereotaxic tau propagation model, AD-PHF injection induced grain-like tau pathology in both wild-type and Picalm+/− mouse brains, but no significant difference in tau pathology was observed between genotypes at injection sites or along the anteroposterior axis. Similarly, working memory performance, assessed by the Y-maze at 3-month-PI, was unaffected by Picalm haploinsufficiency. These results suggest that although *PICALM* expression is modulated by rs3851179 genotype, even a 50% reduction in Picalm expression does not significantly impact tau seeding or propagation in this mouse model. PICALM may thus influence AD risk through mechanisms other than prion-like tau spread [48,91,92]. PICALM, however, is critical in autophagy [91], and mutant tau overexpression combined with reduced Picalm expression causes an alteration in autophagy markers, accelerated tau pathology, and motor deficits in a mouse model of tau pathology, Tg30xPicalm+/− [54]. These data, combined with our present report, suggest that there is a functional dissociation of PICALM between tau spreading and autophagy-mediated tau degradation.

We acknowledge several limitations of this study. First, we analyzed bulk *post-mortem* brain homogenates, which include heterogeneous cell populations. Such analysis provides valuable insights into global gene expression, but it may not fully capture the cell-type-specific regulatory mechanisms that could influence *PICALM* expression, particularly in microglia [93]. Cell-type-specific expression profiling, such as single-cell RNA sequencing or laser capture microdissection, would provide more precise insights into how rs3851179 affects *PICALM* regulation in distinct brain cell types. Second, while we found a significant dissociation between *PICALM* mRNA and protein levels, the mechanistic basis of this discrepancy remains unclear and should be explored through more direct experimental approaches in future work. Third, in our stereotaxic injection model, while the 3-month time point provides valuable insights into the propagation of tau pathology at this stage, it may not fully capture the early or late phases of tau seeding and spreading, which could be influenced by Picalm reduction. We suggest that future studies should incorporate multiple time points to examine the temporal dynamics of tau pathology in more detail, allowing for a more comprehensive understanding of how Picalm may regulate tau propagation across different stages of disease progression. Longer or shorter incubation times could reveal more subtle effects of Picalm haploinsufficiency on tau pathology progression. Lastly, PICALM has been implicated in APP processing via autophagy [91,94] and γ-secretase internalization [95,96]. We were not able to address this aspect here, but future studies should explore how PICALM reduction affects APP metabolism and Aβ clearance.

Despite these limitations, our study provides new insight into the regulatory role of rs3851179 on *PICALM* expression in AD brains, revealing a dissociation between transcript and protein levels that may contribute to disease pathogenesis.

## 5. Conclusions

In conclusion, our data indicate that the AD-protective variant rs3851179T modulates *PICALM* expression at the transcriptional level, whereas this regulation is not proportionally reflected at the protein level in AD brains. The dissociation between *PICALM* mRNA and protein levels, which is influenced by both disease state and genetic background, suggests altered post-transcriptional regulation or protein turnover in AD. Together with our in vivo data showing no major effect of Picalm haploinsufficiency on tau propagation, these findings indicate that *PICALM* likely contributes to AD susceptibility through mechanisms other than tau spreading, such as dysregulated endocytosis, autophagy, or APP processing. Future studies dissecting cell-type-specific and post-transcriptional regulation of PICALM will be crucial to fully understand its protective role in AD.

## Data Availability

The original contributions presented in this study are included in the article/Supplementary Material. Further inquiries can be directed to the corresponding authors. Full-length uncropped WB images are available in Supplementary Figures.

## Supplementary Materials

The following supporting information can be downloaded at: https://www.mdpi.com/article/10.3390/cells15030235/s1, Table S1: Human cases analyzed in this study; Figure S1: Analyses of potential confounding factors and PICALM mRNA expression; Figure S2: Uncropped full images of WB corresponding to Figure 2A; Figure S3: Analyses of potential confounding factors and PICALM protein expression; Figure S4: Uncropped WB images and detailed fractionation workflow; Figure S5: Tau immunohistochemistry in wild-type (WT) and Picalm+/− mice injected with control Sarkosyl-insoluble fractions. Figure S6: Uncropped WB images corresponding to Figure 7A.
