# Supplementary material for: PICALM Genetic Variant Alters mRNA Expression Without Affecting Protein Levels or Tau Spreading in Alzheimer’s Disease
Source: Cells. 2026 Jan 26;15(3):235. doi: 10.3390/cells15030235 (PMC12897012; doi:10.3390/cells15030235)
Supplement: Supplementary file 1 [file cells-15-00235-s001.zip › cells-4030729-supplementary.pdf]

Supplementary Table S1

	Control (n=41)	AD (n=51)
Braak (median +/- SD)	2.00+/-1.39 (n=32)	5.92+/-0.27 (n=51)
Thal (median +/- SD)	1.64+/-1.92 (n=22)	4.36+/-0.87 (n=28)
Sex (% female)	41.46% (n=41)	47.06% (n=48)
Age (median +/- SD)	77.37+/-10.00 (n=41)	75.80+/-10.43 (n=50)
PMD (median +/- SD)	23.33+/-14.20 (n=27)	20.27+/-12.46 (n=47)
ApoE4 (MAF)	0.19 (n=19)	0.29 (n=49)
PICALM rs3851179 (MAF)	0.31 (n=39)	0.28 (n=50)

Table S1. Human cases analyzed in this study.

Neuropathological staging of AD patients was determined according to Braak staging for neurofibrillary tangles (NFTs) [1] and Thal phases for amyloid plaque deposition [2]. The AD cohort includes two familial cases: one carrying the *APP* G2149A mutation and another with *PSEN1* R35E and E120D mutations. Abbreviations: SD, standard deviation; *ApoE,* apolipoprotein E; AD, Alzheimer's disease; PMD, post-mortem delay; MAF, minor allele frequency. Genotyping was performed only in cases with informed consent for genetic analyses. In some individuals, specific information is unavailable due to consent restrictions.

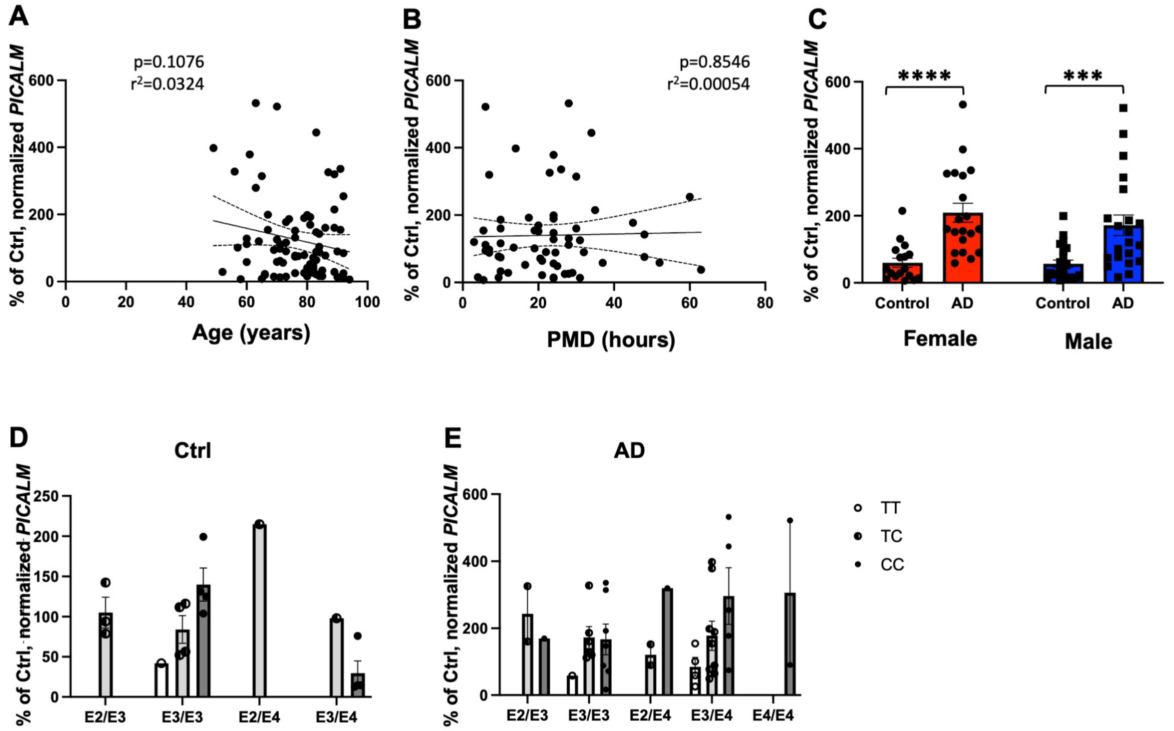

Supplementary Figure S1. Analyses of potential confounding factors and *PICALM* mRNA expression. (**A-B**) No significant correlation was observed between *PICALM* mRNA expression and age (**A**, n=81) or post-mortem delay (PMD) (**B**, n=64) (p>0.05 by Spearman correlation). (**C**) A significant disease effect (AD versus control) was detected, while sex difference had no significant effect on *PICALM* mRNA levels. The disease effect was associated with an increased *PICALM* mRNA level (****p<0.0001, two-way ANOVA). (**D-E**) The non-protective C allele of *PICALM* rs3851179 was generally associated with higher *PICALM* mRNA levels in groups with relatively large sample sizes, including control cases with *ApoE*3/3 (**D**), AD cases with *ApoE*3/3 (**E**), and AD cases with *ApoE*3/4 (**E**).

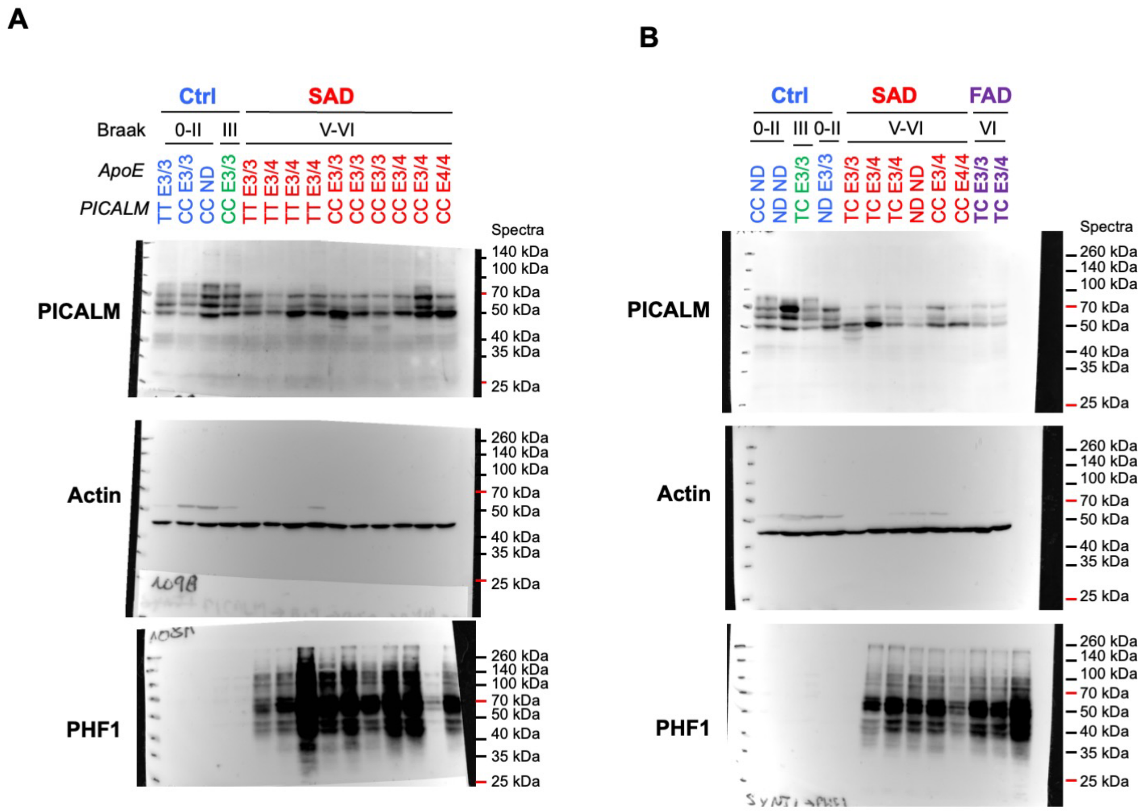

Supplementary Figure S2. Uncropped full images of WB corresponding to Figure 2A.

Brain lysates were analyzed for PICALM, actin and PHF1. Images show merged molecular weight markers (Spectra, Thermo Scientific) and chemiluminescence signals.

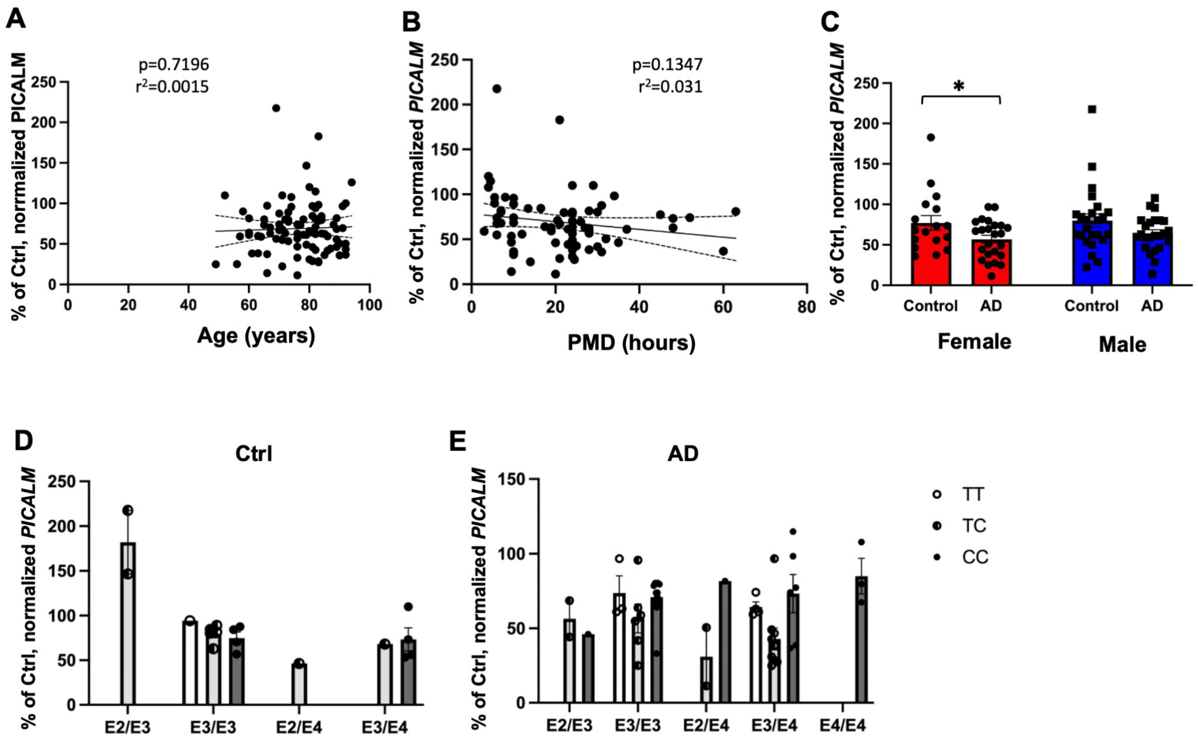

Supplementary Figure S3. Analyses of potential confounding factors and PICALM protein expression. (**A-B**) No significant correlation was observed between PICALM protein expression and age (**A**, n=81) or *post-mortem* delay (PMD) (**B**, n=64) (p>0.05 by Spearman correlation). (**C**) A significant disease effect (AD versus control) was detected, whereas sex difference had no significant effect on PICALM protein levels. The disease effect was associated with decreased PICALM protein expression (**p<0.0092, two-way ANOVA). (**D-E**) The non-protective C allele of *PICALM* rs3851179 was not clearly associated with altered PICALM protein levels even when the cases were grouped according to *ApoE* genotypes.

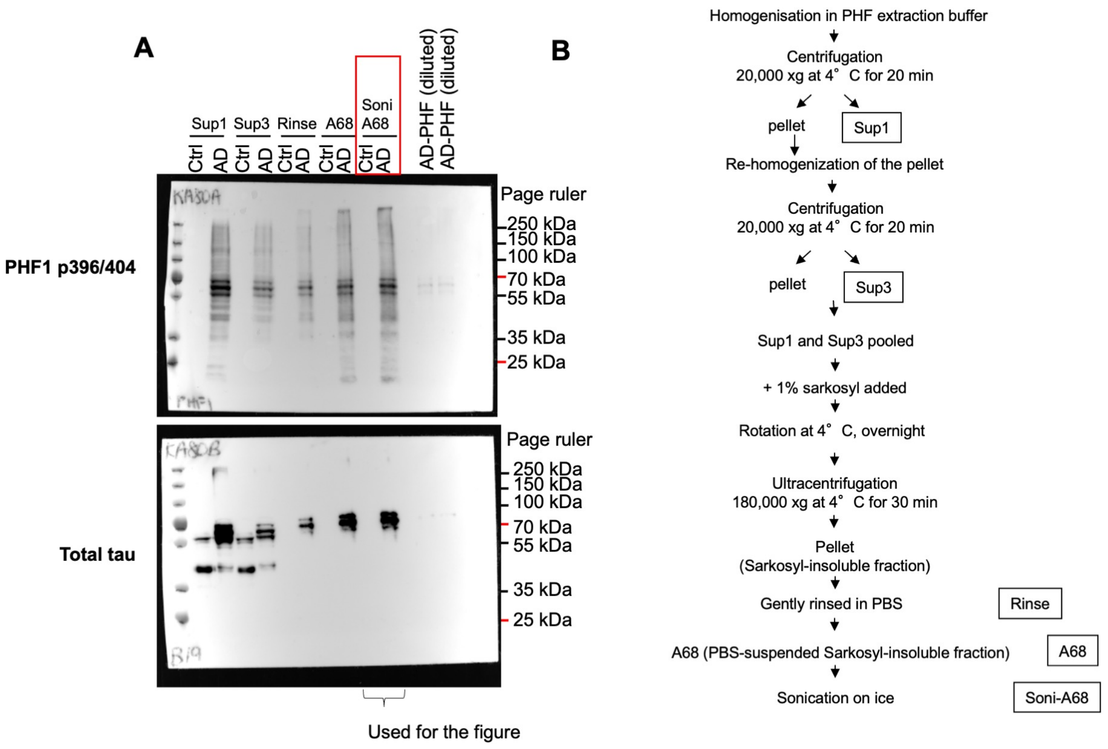

Supplementary Figure S4. Uncropped WB images and detailed fractionation workflow.

(A) Uncropped WB images corresponding to Figure 4B. Different fractions were analyzed for tau using PHF1 antibody (anti-pSer396/Ser404 tau) and anti-total tau B19 antibody. Chemiluminescence signals were merged with the molecular weight markers (Page Ruler, Thermo Fisher). **(B)** Schematic illustration of the steps used to obtain the sonicated Sarkosyl-insoluble fraction (A68) and the corresponding fractions analyzed by WB.

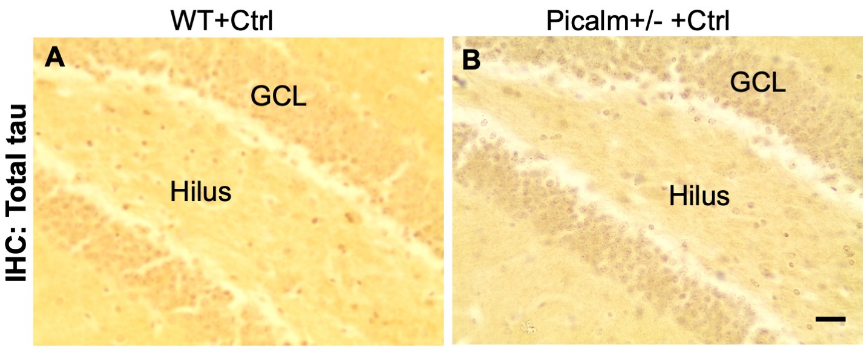

Supplementary Figure S5. Tau immunohistochemistry in wild-type (WT) and Picalm+/- mice injected with control Sarkosyl-insoluble fractions.

(A-B) No tau-positive granular structures were detected in wild-type **(A)** or Picalm+/- **(B)** mice injected with the Sarkosyl-insoluble fraction from a control case. Representative images of the sections stained with rabbit polyclonal anti-total tau (B19) antibody. WT, wild-type; GCL, granular cell layer. A weak hematoxylin counterstaining was applied. Scale bar, 40 µm.

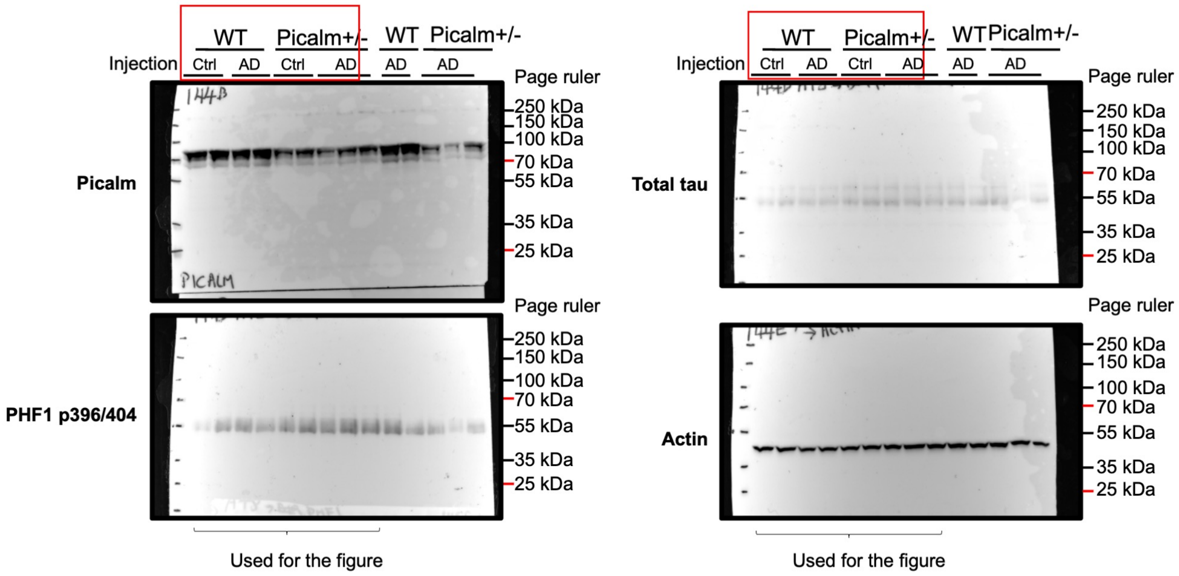

Supplementary Figure S6. Uncropped WB images corresponding to Figure 7A.

Brain lysates from the ipsilateral hemispheres of mice injected with Sarkosyl-insoluble fractions from a control (Ctrl) case or from an AD case (AD-PHF) were analyzed for PICALM, PHF1, total tau (B19) and actin. Chemiluminescence signals were merged with the molecular weight markers (Page Ruler, Thermo Fisher).

References

1 Braak H, Braak E (1991) Neuropathological stageing of Alzheimer-related changes. Acta Neuropathol 82: 239-259 Doi 10.1007/BF00308809

2 Thal DR, Rub U, Orantes M, Braak H (2002) Phases of A beta-deposition in the human brain and its relevance for the development of AD. Neurology 58: 1791-1800
